# Clinical Outcomes of Two Institution-Specific Cervicothoracic Interlaminar Epidural Steroid Injection Protocols (C7/T1–6 mL vs. T1/T2–8 mL) in Cervical Radiculopathy: A Multicenter Retrospective Cohort Study

**DOI:** 10.3390/jcm15155900

**Published:** 2026-07-28

**Authors:** Paweł Gogol, Rafał Pasztaleniec, Robert Szczepaniak, Rafał Wiśniewski, Rafał Staszkiewicz, Michał Sobstyl, Piotr Wąsik, Beniamin Oskar Grabarek

**Affiliations:** 1 Neurolocus Pain Management Centre, 04-281 Warsaw, Poland; drrobertszczepaniak@gmail.com (R.S.); w.rafal@wp.pl (R.W.); 2 Pain Treatment Clinic, Our Lady of Perpetual Help Hospital in Wołomin, 05-200 Wołomin, Poland; 3 Department of Neurosurgery, Faculty of Medicine, Academy of Silesia, 40-555 Katowice, Poland; rafalstaszkiewicz830@gmail.com; 4 Pain Medicine Centre, 64-920 Piła, Poland; drrafalpasztaleniec@gmail.com; 5 Department of Neurosurgery, Institute of Psychiatry and Neurology, 02-957 Warsaw, Poland; mrsob@op.pl; 6 Pain Management Clinic, St. Luke’s Hospital in Bolesławiec, 59-700 Bolesławiec, Poland; pio.wasik@gmail.com; 7 Pain Management Clinic, District Hospital in Czarnków, 64-700 Czarnków, Poland; 8 Collegium Medicum, WSB University, 41-300 Dąbrowa Górnicza, Poland; bgrabarek7@gmail.com

**Keywords:** cervical radiculopathy, epidural steroid injection, interlaminar approach, cervicothoracic junction, fluoroscopy, pain management, neck disability index, corticosteroid safety

## Abstract

**Background:** Cervical radiculopathy is a common and disabling condition with both nociceptive and neuropathic pain components. Epidural steroid injections (ESIs) are widely used as an interventional treatment modality; however, the optimal cervicothoracic interlaminar access level and injectate volume remain subjects of ongoing debate. Because injection level and injectate volume have historically been varied together in routine practice rather than independently, prospectively designed comparative data are lacking. **Methods:** In this retrospective, multicenter cohort study, 166 patients with MRI-confirmed cervical radiculopathy treated between January 2022 and December 2025 at three participating institutions received fluoroscopy-guided cervicothoracic interlaminar epidural steroid injections with dexamethasone and bupivacaine at either C7/T1 (6 mL; *n* = 87) or T1/T2 (8 mL; *n* = 79), with protocol determined by the treating institution rather than randomization. The prespecified primary outcome was the baseline-adjusted between-group difference in arm-pain Numeric Rating Scale (NRS) score at 3 months, analyzed by analysis of covariance (ANCOVA) adjusted for a directed acyclic graph-informed covariate set (age, symptom duration, baseline pain severity, number of stenotic levels, DN4 score, and treatment center); the same covariates were used in a generalized estimating equation (GEE) model with a group-by-time interaction and in a multivariable logistic regression for treatment response. Secondary outcomes—neck-pain NRS and VAS, neuropathic pain (DN4), functional disability (NDI), quality of life (SF-36, EQ-5D), Patient Global Impression of Change (PGIC), analgesic consumption, and cardiovascular/metabolic safety outcomes—were assessed at baseline, 3 months, and 6 months. **Results:** Both groups showed significant within-group reductions in arm-pain NRS (C7/T1: 8.7 ± 0.4 to 3.5 ± 0.4 at 3 months and 4.1 ± 0.6 at 6 months; T1/T2: 9.2 ± 0.7 to 3.9 ± 0.5 at 3 months and 4.9 ± 0.7 at 6 months; both *p* < 0.001). Baseline pain scores and symptom duration were not balanced between groups (baseline-adjusted analysis was therefore used). After adjustment for baseline severity, age, symptom duration, and center, the T1/T2–8 mL protocol was associated with higher (worse) adjusted arm-pain NRS than the C7/T1–6 mL protocol at 3 months (adjusted difference 0.41 points, 95% CI 0.21–0.61) and 6 months (0.66 points, 95% CI 0.35–0.97); a multivariable-adjusted logistic regression model likewise showed lower odds of achieving a ≥50% arm-pain reduction at 3 months with the T1/T2–8 mL protocol (adjusted OR 0.18, 95% CI 0.04–0.82). NDI decreased from a mean of 48.1 points at baseline to 28.1 points at 3 months (pooled cohort, score capped at the 0–50 scale maximum; the majority of baseline NDI source records could not be individually verified and this is reported as a limitation), and analgesic consumption decreased over follow-up. Among hypertensive patients (45/166, 27.1%), 78% experienced transient blood pressure elevation and 40% required temporary antihypertensive adjustment; among diabetic patients (13/166, 7.8%), 92% developed transient hyperglycemia. Because injection level, injectate volume, and treatment center were confounded by design, these findings should be interpreted as a comparison of two center-specific treatment protocols rather than an isolated effect of anatomical level or volume. **Conclusions:** Both center-specific cervicothoracic interlaminar epidural steroid injection protocols were associated with improvements in pain and function over six months, with no catastrophic procedural complications documented; however, near-complete confounding of injection level with injectate volume and treatment center, together with imbalanced baseline pain severity, precludes conclusions about the comparative effectiveness of C7/T1 versus T1/T2 access. Transient cardiovascular and metabolic effects were common and support consideration of post-procedural monitoring in patients with pre-existing hypertension or diabetes.

## 1. Introduction

Cervical radiculopathy is a clinical syndrome resulting from compression and inflammation of cervical nerve roots, most commonly due to degenerative changes such as intervertebral disc herniation, osteophyte formation, uncovertebral hypertrophy, and foraminal stenosis [1,2]. The condition is characterized by neck pain radiating into the upper extremity, often accompanied by paresthesia, sensory disturbances, reduced cervical mobility, muscle spasm, and, in some cases, motor deficits.

The pathophysiology of cervical radiculopathy involves both mechanical compression and inflammatory biochemical processes. Nerve root irritation results in the release of inflammatory mediators, edema formation, and sensitization of nociceptive pathways. Consequently, pain associated with cervical radiculopathy is frequently multifactorial, involving both nociceptive and neuropathic mechanisms [3]. This mixed pain phenotype contributes to the complexity of treatment and often necessitates multimodal management strategies.

Conservative treatment typically includes nonsteroidal anti-inflammatory drugs (NSAIDs), neuropathic pain medications, physiotherapy, rehabilitation, and activity modification. However, a considerable proportion of patients continue to experience significant pain and disability despite prolonged conservative management.

Epidural steroid injections (ESIs) are widely used as an intermediate step between conservative therapy and surgical intervention. Their primary mechanism involves the anti-inflammatory effects of corticosteroids, which reduce perineural edema and inhibit inflammatory mediators [2,4,5]. ESIs may also exert secondary effects on nociceptive transmission and central sensitization.

The interlaminar approach is commonly favored in the cervical region due to a lower risk profile compared with the transforaminal approach, which has been associated with rare but catastrophic complications such as spinal cord infarction, vertebral artery injury, and embolic ischemic events [6,7,8,9,10]. The cervicothoracic junction, particularly the C7/T1 and T1/T2 interspaces, is frequently selected because of its relatively wider epidural space and technically favorable anatomy.

Despite the widespread use of interlaminar ESIs, several technical aspects remain debated, including the optimal injection level at the cervicothoracic junction and the influence of injectate volume on clinical outcomes. Comparative data regarding C7/T1 versus T1/T2 injections are limited, and in routine practice injection level and injectate volume are typically varied together rather than independently, so their individual contributions to outcome remain unclear. One randomized trial comparing a targeted epidural catheter technique with a standard C7/T1 interlaminar approach for unilateral cervical radicular pain found no significant difference in clinical outcomes between techniques, and studies of contrast spread at the cervicothoracic junction indicate that both the injected volume and needle-tip position materially affect cephalad and ventral distribution of injectate, which has in turn been correlated with clinical outcome in some series; current evidence-based guidelines from the American Academy of Neurology and the North American Spine Society summarize the overall efficacy data for cervical epidural steroid injections but do not make a specific recommendation regarding injection level or volume at the cervicothoracic junction. This study was exploratory rather than confirmatory: we did not hypothesize a priori that either protocol would be superior, and the analysis was not designed or powered as an equivalence or non-inferiority comparison [11,12,13].

The aim of this retrospective, multicenter cohort study was to describe clinical outcomes and safety associated with two center-specific cervicothoracic interlaminar epidural steroid injection protocols (C7/T1 with a 6 mL injectate volume and T1/T2 with an 8 mL injectate volume) in patients with cervical radiculopathy, with particular emphasis on pain reduction, functional improvement, medication use, and systemic effects of corticosteroid administration. Because treatment protocol was assigned according to treating institution rather than randomized, and injection level and injectate volume varied together, this study compares two institutional treatment protocols rather than isolating the independent effect of anatomical access level or volume. Although this comparison cannot isolate anatomical access level from injectate volume, multicenter, real-world comparative data on cervicothoracic interlaminar access-level protocols that combine multiple validated pain, functional, and quality-of-life instruments with an adjusted, DAG-informed safety and effectiveness comparison remain scarce in the published literature; this study is intended to complement, rather than replace, smaller controlled trials by providing such comparative institutional-protocol data from routine clinical practice.

## 2. Materials and Methods

### 2.1. Study Design and Setting

This retrospective multicenter study was conducted between January 2022 and December 2025 at three institutions specializing in interventional pain management: Neurolocus Pain Management Centre; Blessed Virgin Mary Perpetual Help Hospital in Wołomin; and R. Surma District Hospital in Czarnków. The study protocol was based on standardized interventional pain management procedures routinely implemented at the participating centers, and all procedures were performed by experienced pain medicine specialists with extensive experience in fluoroscopy-guided spine interventions. The study was performed in accordance with the guidelines of the 2013 Declaration of Helsinki on human experimentation, and data confidentiality and patient anonymity were maintained at all times throughout data collection, storage, and analysis. All patients provided informed consent for the epidural steroid injection procedure itself at the time of treatment, in accordance with routine clinical practice; because the present analysis was retrospective and based on fully anonymized data, the Bioethical Committee of the Regional Medical Chamber in Kraków waived the requirement for additional, study-specific research consent. A single ethical approval (No. 89/KBL/OIL/2026, dated 10 March 2026) covered the retrospective review and analysis of clinical data from all three participating centers for the entire study period (2022–2025); data extraction and statistical analysis were performed only after this approval had been obtained.

### 2.2. Study Population and Eligibility Criteria

**Figure 1:**
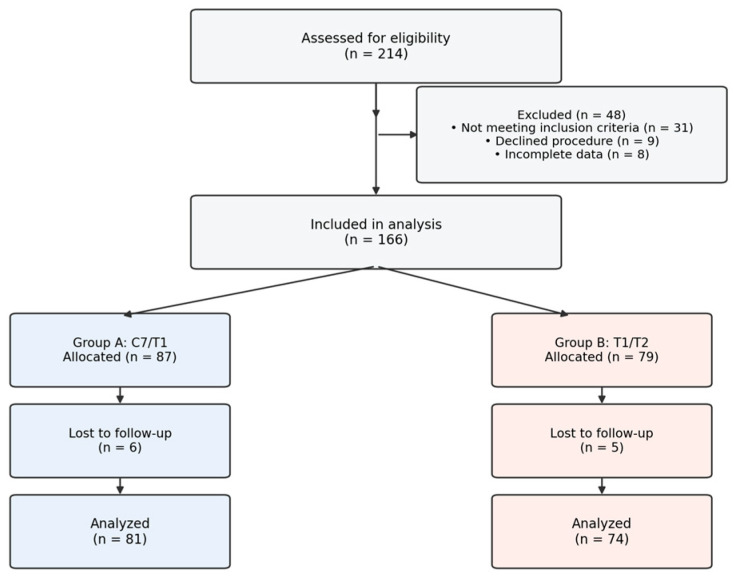
STROBE-style flow diagram illustrating patient enrollment, allocation, and follow-up in the two treatment groups [14]. *n*: number of patients.

A total of 166 patients aged 18–65 years were included in the study (Figure 1). Eligible patients had MRI-confirmed cervical radiculopathy with foraminal stenosis involving one or two cervical levels, unilateral or bilateral radicular symptoms of at least six weeks’ duration, and an inadequate response to a course of conservative therapy comprising NSAIDs, physiotherapy, and structured rehabilitation. All included patients had preserved motor strength without a clinically significant motor deficit and had no contraindication to corticosteroid administration. Patients were identified retrospectively from institutional records of those who had already undergone injection during the study period; cohort assembly and the criteria for consecutive inclusion are described in Section 2.1 and Figure 1. Patients were excluded from the study if they presented with a progressive neurological deficit, cervical myelopathy, active infection, active malignancy, unexplained weight loss, fever, recent major trauma, bowel or bladder dysfunction, coagulopathy, ongoing anticoagulant therapy incompatible with the procedure, a platelet count below 100,000/µL, a known allergy to any of the study medications, a history of neurodegenerative or demyelinating disease, or severe central spinal stenosis carrying a risk of injectate loculation. All patients had undergone prior neurosurgical consultation and were judged to be candidates for conservative or interventional management rather than immediate surgery. Patients were allocated according to the interlaminar level used at the treating institution: Group A (C7/T1) comprised 87 patients (46 men, 41 women), and Group B (T1/T2) comprised 79 patients (42 men, 37 women). Patients presented predominantly with radicular neck pain radiating to the upper limb, paresthesia, sensory disturbances, reduced cervical mobility, and a clinically significant neuropathic pain component. Baseline demographic and clinical characteristics, which were comparable between the two groups, are summarized in Table 1.

### 2.3. Procedure

All procedures were performed under strict aseptic conditions using fluoroscopic guidance. Patients were positioned prone on the fluoroscopy table with the neck slightly flexed to widen the target interlaminar space. Following standard skin disinfection and sterile draping, local anesthesia was infiltrated using 2 mL of 2% lidocaine (lidocaine hydrochloride; Fresenius Kabi Polska Sp z o. o., Warsaw, Poland). A Tuohy needle was introduced via a midline interlaminar approach at the target level under anteroposterior fluoroscopic guidance. The epidural space was identified using the loss-of-resistance (LOR) technique with saline, and correct needle placement was confirmed by injection of a non-ionic contrast medium under real-time fluoroscopy in anteroposterior and lateral projections, demonstrating a typical epidurogram without vascular or subarachnoid spread. The injectate consisted of dexamethasone sodium phosphate 12 mg (dexamethasone; Polpharma Warszawa S.A., Warsaw, Poland) combined with 0.125% bupivacaine (bupivacaine hydrochloride; Polpharma Warszawa S.A., Warsaw, Poland). Patients allocated to the C7/T1 level received a total injectate volume of 6 mL, while those allocated to the T1/T2 level received a total volume of 8 mL, in accordance with the standard institutional protocol at the participating centers. Following the procedure, all patients were monitored for a minimum of 60 min for immediate procedural complications, including hemodynamic instability, neurological changes, or clinical signs suggestive of intrathecal spread.

### 2.4. Outcome Measures

Pain intensity was assessed using two complementary self-report instruments. The Numeric Rating Scale (NRS) is an 11-point scale ranging from 0 (“no pain”) to 10 (“worst pain imaginable”) on which the patient selects the integer that best represents current pain intensity [15]. The Visual Analog Scale (VAS) consists of a 100-mm horizontal line anchored by “no pain” (0 mm) and “worst imaginable pain” (100 mm), on which the patient marks the point corresponding to perceived pain intensity, subsequently measured in millimeters from the left anchor [16]. Both instruments are extensively validated in musculoskeletal and radicular pain populations and provide complementary, strongly correlated information on pain severity.

Neuropathic pain was evaluated using the DN4 (Douleur Neuropathique en 4 Questions) questionnaire, a validated 10-item tool combining seven interview-based items—assessing burning sensation, painful cold, electric shocks, tingling, pins-and-needles sensation, numbness, and itching—with three items derived from bedside sensory examination (hypoesthesia to touch, hypoesthesia to pinprick, and touch-evoked allodynia); a total score of 4 or more out of 10 is considered indicative of a neuropathic pain component [17].

Functional disability related to neck pain was assessed with the Neck Disability Index (NDI), a 10-item, patient-completed questionnaire evaluating pain intensity and its impact on personal care, lifting, reading, headaches, concentration, work, driving, sleep, and recreation; each item is scored from 0 to 5, yielding a total score ranging from 0 to 50 (or expressed as a percentage), with higher scores indicating greater disability [18].

Health-related quality of life was measured using two complementary instruments. The Short Form-36 Health Survey (SF-36) yields two summary scores, the Physical Component Summary (PCS) and Mental Component Summary (MCS), each normalized to a 0–100 scale on which higher scores reflect better health status [19]. The EuroQol 5-Dimension (EQ-5D) instrument describes health status across five domains—mobility, self-care, usual activities, pain/discomfort, and anxiety/depression—and generates a single utility index anchored at 0 (equivalent to death) and 1 (full health) [20].

Global treatment response was captured with the Patient Global Impression of Change (PGIC), a single-item, 7-point scale ranging from “very much worse” to “very much improved,” which is widely used to contextualize statistically significant changes on disease-specific instruments against the patient’s own perception of clinically meaningful improvement [21].

At each visit, outcome instruments were administered by the treating physician, a dedicated study coordinator, or a research nurse, depending on center and visit type; the source clinical records did not document a formal, standardized inter-rater training or certification protocol for outcome assessment across the three centers, and this is noted as a limitation.

Analgesic consumption was recorded from prescription and pharmacy records and patient diaries. NSAID use included diclofenac (50–150 mg/day; Novartis Pharma AG, Basel, Switzerland), ketoprofen (50–200 mg/day; Sandoz d.d., Ljubljana, Slovenia), and ibuprofen (400–1600 mg/day; Reckitt Benckiser Healthcare Ltd., Slough, UK). Opioid-containing analgesia consisted of fixed-dose combinations of tramadol with paracetamol (Grünenthal GmbH, Aachen, Germany), and tramadol with dexketoprofen (Menarini International Operations Luxembourg S.A., Luxembourg CityLuxembourg). Safety outcomes comprised procedural complications, cardiovascular effects (blood pressure changes and the need for antihypertensive adjustment), and metabolic effects (blood glucose changes and the need for glycemic treatment adjustment), all recorded at scheduled follow-up visits and through review of outpatient records.

Operational thresholds for the transient hemodynamic and metabolic events reported in Section 3.6 were: mild hypotension, a systolic blood pressure (SBP) decrease of ≥20 mmHg from the pre-procedure baseline or an absolute SBP <90 mmHg; transient blood pressure elevation, an SBP increase of ≥20 mmHg from baseline or an absolute SBP ≥160 mmHg; and transient hyperglycemia, a fasting glucose ≥126 mg/dL or an increase of ≥30 mg/dL from baseline. Dizziness/lightheadedness and transient upper-limb numbness were recorded when spontaneously reported or elicited on direct questioning; transient disorientation was recorded when observed or reported without loss of consciousness. Concurrent pharmacological treatment during follow-up is captured through the analgesic-consumption data reported in Section 3.4; concurrent non-pharmacological treatment (e.g., physiotherapy continued or resumed during the 6-month follow-up period) was not systematically recorded in the source clinical documentation beyond the pre-injection conservative-management history described in Section 2.2, and this is noted as a limitation.

### 2.5. Statistical Analysis

Statistical analysis was performed using StatPlus software (StatPlus Pro v7.3, AnalystSoft Inc., Brandon, FL, USA). Continuous variables were expressed as mean ± standard deviation, and normality of distribution was assessed using the Shapiro–Wilk test together with graphical inspection (Q–Q plots) and residual diagnostics rather than the Shapiro–Wilk test alone. Baseline between-group comparisons were performed using Student’s *t*-test or the Mann–Whitney U test as appropriate, and standardized mean differences were additionally calculated because *p*-values for baseline comparisons are of limited interpretive value in a non-randomized design. Because injection level, injectate volume, and treatment center were not fully separable (two of the three centers used both protocols, while the third used only the T1/T2–8 mL protocol; Table S1), a directed acyclic graph (DAG) was constructed a priori from the interventional pain literature to identify variables acting as confounders of the association between treatment protocol and outcome, comprising age, symptom duration, baseline pain severity, number of stenotic levels, neuropathic pain component (DN4), and treatment center; these variables were entered as covariates in adjustment models rather than variables considered to be mediators of treatment. Repeated-measures continuous outcomes (NRS, VAS, NDI, SF-36, EQ-5D, DN4) were analyzed using baseline-adjusted linear models (ANCOVA) at each follow-up time point and, for the primary outcome, a generalized estimating equation (GEE) with an exchangeable working correlation structure to model the protocol-by-time interaction, with age, symptom duration, and center as covariates. Binary treatment-response outcomes (e.g., ≥50% reduction in arm-pain NRS at 3 months) were analyzed using multivariable logistic regression adjusted for the same DAG-selected covariates, reported as adjusted odds ratios with 95% confidence intervals. All between-group estimates are reported as adjusted mean differences or odds ratios with 95% confidence intervals in addition to *p*-values. Because protocol was strongly, though not perfectly, correlated with treatment center, the independent effects of injection level, injectate volume, and center could not be fully disentangled, and this is discussed as a limitation. Because this was a retrospective study in which all consecutive eligible patients treated during the study period were included, no prospective sample-size calculation was performed. A post-hoc analysis of the precision achievable with the observed sample size and variability was performed for the primary outcome in place of a traditional post-hoc power calculation, given recognized concerns about the interpretability of post-hoc power estimates; the width of the resulting 95% confidence intervals is reported alongside the minimum between-group difference detectable with 80% power at α = 0.05. No formal correction for multiplicity was applied across the full set of secondary and exploratory outcomes; consequently, secondary and safety analyses should be interpreted as hypothesis-generating, and only the pre-specified primary outcome (adjusted between-group difference in arm-pain NRS at 3 months) supports confirmatory interpretation. Missing data are described by outcome and time point in Section 3.1 and Table S3; primary analyses used all available data under a missing-at-random assumption (GEE/available-case), and reasons for loss to follow-up are reported by group. Categorical variables were compared using the χ^2^ test or Fisher’s exact test where expected cell counts were small, and exact (Wilson) 95% confidence intervals are reported for proportions based on small subgroups (e.g., diabetes, *n* = 13). A *p*-value below 0.05 was considered statistically significant for the primary outcome.

## 3. Results

### 3.1. Patient Flow and Baseline Characteristics

Of 214 patients assessed for eligibility, 48 were excluded (31 not meeting inclusion criteria, 9 declined the procedure, and 8 with incomplete data), leaving 166 patients for analysis (Figure 1); reasons for non-inclusion by center are given in Table S2. Six patients in Group A and five in Group B were lost to follow-up, yielding 81 and 74 patients analyzed at final follow-up, respectively (Table S3). Baseline demographic and clinical characteristics are summarized in Table 1. Corrected between-group comparisons (see Section 2.5) showed that symptom duration and baseline arm-pain severity were not balanced between groups, whereas age, sex distribution, body mass index, extent of foraminal stenosis, and the prevalence of hypertension or diabetes mellitus did not differ significantly (Table 1). Because of this baseline imbalance, all between-group outcome comparisons reported below use baseline-adjusted estimates rather than raw between-group differences.

### 3.2. Pain Outcomes

**Figure 2:**
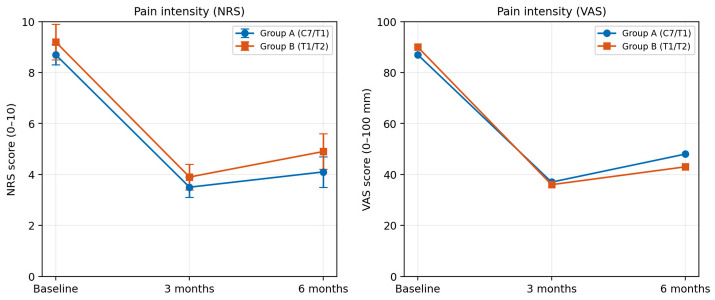
Mean pain intensity over time by treatment group, expressed as NRS (**left**) and VAS (**right**) scores. Error bars represent standard deviation. NRS: Numeric Rating Scale; VAS: Visual Analog Scale.

Both groups demonstrated significant reductions in pain scores. In Group A, the NRS decreased from 8.7 ± 0.4 at baseline to 3.5 ± 0.4 at 3 months and 4.1 ± 0.6 at 6 months; in Group B, the NRS decreased from 9.2 ± 0.7 at baseline to 3.9 ± 0.5 at 3 months and 4.9 ± 0.7 at 6 months (*p* < 0.001 for both groups, Table 2, Figure 2). A parallel pattern was observed for VAS scores, which decreased from 87 ± 4 mm to 37 ± 4 mm at 3 months and 48 ± 7 mm at 6 months in Group A, and from 90 ± 8 mm to 36 ± 5 mm at 3 months and 43 ± 2 mm at 6 months in Group B (*p* < 0.001). Unadjusted between-group comparisons were statistically significant at most timepoints for both instruments, and baseline-adjusted analyses (Section 2.5) showed a higher (worse) adjusted arm-pain NRS in the T1/T2–8 mL group at 3 months (adjusted difference 0.41 points, 95% CI 0.21–0.61) and 6 months (0.66 points, 95% CI 0.35–0.97); the corresponding VAS-based adjusted differences were not statistically significant at 3 months and, unlike the NRS-based estimates, favored the T1/T2–8 mL protocol (i.e., pointed in the opposite direction) by 6 months; this NRS-VAS discordance for arm and neck pain at 6 months could not be resolved with the available data and is discussed as a limitation rather than reconciled post hoc. Because injection level, injectate volume, and center were not fully separable, these differences cannot be attributed uniquely to anatomical level.

### 3.3. Functional and Quality-of-Life Outcomes

Functional outcomes improved significantly across the pooled cohort (Table 3). The NDI decreased from 48.1 ± 5.4 points at baseline (capped at the 0–50 scale maximum; source records for the majority of baseline NDI values could not be individually re-verified, see Limitations) to 28.1 ± 4.5 points at 3 months and 31.4 ± 4.6 points at 6 months (*p* < 0.001). SF-36 Physical Component Summary scores improved from 32.6 ± 2.7 to 47.2 ± 4.1 at 3 months, and Mental Component Summary scores improved from 35.9 ± 4.5 to 52.7 ± 5.2. EQ-5D index scores improved from 0.46 ± 0.09 to 0.75 ± 0.03 at 3 months. DN4 scores decreased from 6.2 ± 1.4 to 2.2 ± 0.7 at 3 months, reflecting a marked reduction in the neuropathic pain component (Table 3).

### 3.4. Analgesic Consumption

NSAID consumption decreased from a mean of 116.4 DDD-equivalent units at baseline to approximately 41 units at 3 months and 70 units at 6 months (pooled cohort); opioid consumption (morphine milligram equivalents, MME) decreased from a mean of 12.8 mg/day at baseline to approximately 5.1 mg/day at 3 months and 6.4 mg/day at 6 months. Baseline opioid consumption was higher in the C7/T1 group than the T1/T2 group (15.3 vs. 9.9 mg/day, *p* = 0.007), and absolute (not only percentage) values by group are reported in Table 4 for this reason.

### 3.5. Patient Global Impression of Change

At 3 months, 70% of patients reported being much improved or very much improved, 20% reported moderate improvement, and 10% reported minimal or no improvement; at 6 months, the corresponding proportions were 60%, 20%, and 20% (Table 5). Group-specific counts and denominators are reported in Table 5 rather than pooled percentages alone.

### 3.6. Adverse Events and Safety

**Figure 3:**
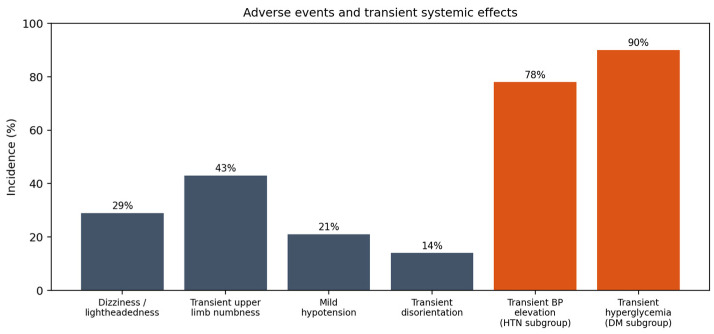
Incidence of adverse events and transient cardiovascular/metabolic effects across the study population. BP: blood pressure; HTN: hypertension; DM: diabetes mellitus.

Common adverse effects included dizziness/lightheadedness (30%; 26% in Group A, 34% in Group B), transient upper-limb numbness (41%; 40% vs. 42%), mild hypotension (16%; 20% vs. 11%), and transient disorientation (13%; 9% vs. 18%); overall percentages here are recalculated directly from event counts and denominators (Table 6) and differ slightly from the originally reported overall rates, which were not internally consistent with the group-specific rates. None of the between-group differences reached statistical significance, and no severe neurological or vascular complications were observed in either group; however, with 166 patients this study cannot exclude rare serious complications (Table 6, Figure 3).

### 3.7. Cardiovascular and Metabolic Effects

Among the entire study population, 45 patients (27.1%) had pre-existing hypertension. Within this subgroup, 35/45 (77.8%, 95% CI 63.7–87.5%) experienced transient elevation of blood pressure within 5 days following the injection, and 18/45 (40.0%) required temporary adjustment of antihypertensive therapy; no hypertensive emergencies or hospitalizations occurred. Thirteen patients (7.8%) had diabetes mellitus; within this subgroup, 12/13 (92.3%, 95% CI 66.7–98.6%) experienced transient hyperglycemia lasting up to 7 days, and 8/13 (61.5%) required additional hypoglycemic medication or an increased insulin dosage. No episodes of diabetic ketoacidosis or hospitalization were reported (Table 7). Given the small subgroup sizes, particularly for diabetes (*n* = 13), these estimates should be interpreted with the exact confidence intervals reported here rather than as precise population rates.

### 3.8. Surgical Outcomes

At final follow-up, 16% of patients underwent surgical treatment, with no statistically significant difference between groups.

### 3.9. Adjusted and Multivariable Analyses

Baseline-adjusted comparisons (ANCOVA), group × time interactions (GEE), and the multivariable logistic regression model for treatment response are reported directly in Table 8, in addition to their description in Section 2.5. For arm- and neck-pain NRS, the T1/T2–8 mL protocol was associated with a small but statistically significant adjusted disadvantage at both 3 and 6 months, consistent with the responder analysis (adjusted OR for achieving ≥50% arm-pain reduction at 3 months = 0.18, 95% CI 0.04–0.82, *p* = 0.026). In contrast, VAS-based adjusted differences for arm and neck pain were not statistically significant at 3 months and pointed in the opposite direction at 6 months; this NRS–VAS discordance could not be resolved with the data available and is discussed as a limitation rather than reconciled post hoc. Adjusted differences for NDI were not statistically significant at either timepoint, consistent with the baseline NDI data-quality concerns described in Section 3.3. The same baseline-adjusted ANCOVA approach was also applied to the remaining repeated-measures secondary outcomes (SF-36 PCS, SF-36 MCS, EQ-5D index, and DN4); none showed a statistically significant between-group difference at 3 or 6 months (Table 8), indicating that the pattern of improvement in general health status, quality of life, and neuropathic pain burden did not differ materially between protocols once baseline values and covariates were accounted for.

## 4. Discussion

In this multicenter retrospective cohort, cervicothoracic interlaminar epidural steroid injections were followed by significant improvements in pain, functional status, and quality of life in patients with cervical radiculopathy; as an uncontrolled study, this cannot establish that the injections caused these improvements. The magnitude of pain reduction and functional improvement observed in this cohort is consistent with previously published systematic reviews and meta-analyses evaluating epidural steroid injections in radicular pain syndromes [22,23]. Importantly, the observed benefit was consistent across multiple validated outcome instruments, including the NRS, VAS, NDI, SF-36, EQ-5D, and DN4, suggesting a robust and multidimensional therapeutic effect that extends beyond simple analgesia to encompass function, psychological wellbeing, and health-related quality of life. Two recent studies provide additional context for these findings: a randomized controlled trial found that adding a structured neck-stabilization exercise program after cervical interlaminar ESI produced greater and more sustained improvement than injection alone, suggesting that the benefits observed in the present cohort, achieved without a standardized post-injection exercise protocol, may be conservative relative to what could be achieved with adjunctive rehabilitation; and a separate retrospective cohort using Patient-Reported Outcomes Measurement Information System (PROMIS) physical function and pain interference domains similarly reported significant improvement after cervical interlaminar ESI, supporting the generalizability of the present findings across different outcome measurement frameworks [24,25].

The unadjusted comparison between injections performed at the C7/T1 and T1/T2 protocols did not show a consistent pattern of differences across all instruments, but the baseline-adjusted analysis (Section 2.5 and Section 3.2) showed a statistically significant, though modest, disadvantage for the T1/T2–8 mL protocol on arm-pain NRS at 3 and 6 months. Because injection level, injectate volume, and treatment center were not independently identifiable in this dataset, this finding cannot be attributed specifically to anatomical entry point, injectate volume, or institutional practice, and should not be interpreted as evidence that either protocol is superior. The suggestion that epidural spread of injectate at the cervicothoracic junction is sufficient to achieve comparable clinical outcomes regardless of entry point is a plausible hypothesis but is speculative in the absence of measured cephalad contrast spread data in this cohort, and should be labelled as such rather than presented as an established mechanism. Likewise, the assertion that the modest difference in injectate volume between the two protocols (6 mL vs. 8 mL) did not influence outcomes is not supportable, because volume was inseparable from injection level and center in this dataset; prior evidence on volume-dependent cephalad spread [26,27] indicates that injectate volume materially affects the extent of cephalad and ventral epidural spread and should therefore be regarded as a relevant, uncontrolled variable in the present comparison rather than one that can be assumed not to matter. Consequently, these findings do not support treating injection level or injectate volume as interchangeable based on operator or institutional preference alone; independent confirmation, ideally in a design that varies level and volume separately within the same center, would be needed before such a recommendation could be made.

The choice of dexamethasone as the corticosteroid component in this protocol also merits comment. Dexamethasone is a non-particulate steroid, and its use in the cervical region has been increasingly favored over particulate formulations (such as triamcinolone or methylprednisolone acetate) because particulate steroids carry a theoretical risk of vascular embolization and catastrophic neurological injury if inadvertent intra-arterial injection occurs, a concern that has driven regulatory and specialty-society caution regarding transforaminal cervical injections in particular [28]. While the interlaminar approach used in the present study is inherently associated with a lower risk of intravascular injection than the transforaminal route, the additional use of a non-particulate agent likely contributed further to the favorable safety profile observed, with no severe neurological or vascular complications recorded in either treatment group.

The significant reduction in DN4 scores highlights the importance of targeting the neuropathic component of cervical radiculopathy, which is frequently underrecognized in clinical practice when pain is assessed using unidimensional nociceptive scales alone. This finding aligns with the contemporary understanding that radicular pain represents a mixed pain state incorporating both nociceptive and neuropathic elements, and it reinforces the rationale for multimodal management strategies that combine, where indicated, structural interventions directed at the compressive pathology with adjunctive pharmacological and non-pharmacological neuropathic pain management [3,29,30].

An important contribution of this study is the detailed evaluation of systemic effects associated with epidural corticosteroid administration, an aspect that is frequently underreported in the interventional spine literature relative to procedural complications. Among patients with pre-existing hypertension, 78% experienced transient elevations in blood pressure, and 40% required temporary adjustment of antihypertensive therapy; similarly, among patients with diabetes mellitus, transient hyperglycemia was observed in 90%, with 60% requiring pharmacological intervention. These findings are consistent with previous reports demonstrating measurable systemic metabolic effects following epidural steroid injections, which arise from partial systemic absorption of the corticosteroid from the epidural space into the systemic circulation, with resultant transient suppression of endogenous cortisol production and interference with glycemic and vascular homeostasis [31,32]. Although these effects were uniformly transient and did not result in severe complications such as hypertensive emergency or diabetic ketoacidosis, they are clinically significant and should not be underestimated, particularly given the high prevalence of cardiovascular and metabolic comorbidities in patients presenting for interventional pain management.

From a regulatory and safety perspective, the interlaminar approach used in this study demonstrated a favorable risk profile, with only mild and transient adverse events observed and no severe neurological or vascular complications. This is consistent with prior literature indicating that interlaminar injections are associated with substantially lower rates of catastrophic complications compared with transforaminal techniques, which have been the subject of specific regulatory risk communications following reports of spinal cord infarction and other serious neurological events [33,34]. Taken together with the present findings, these data support the continued preference for the interlaminar route at the cervicothoracic junction in routine clinical practice, particularly when a non-particulate steroid is used.

The results of this study are broadly concordant with current clinical practice guidelines for the management of neck pain and cervical radiculopathy, which recommend a stepwise approach progressing from conservative management to interventional techniques such as epidural steroid injection prior to consideration of surgery in appropriately selected patients [2,35]. The 16% surgical conversion rate observed in this cohort at final follow-up is consistent with contemporary reviews reporting that the majority of patients with cervical radiculopathy who respond to interventional pain management can avoid surgical intervention, at least in the short-to-medium term [36].

This study has several important limitations, some of which are fundamental rather than editorial. Most importantly, injection level (C7/T1 vs. T1/T2), injectate volume (6 mL vs. 8 mL), and treatment center were confounded by design: two of three centers used both protocols and one center used only the T1/T2–8 mL protocol, so the independent effects of level, volume, center, and operator cannot be fully disentangled, and this study should be understood as a comparison of two center-specific treatment protocols rather than an isolated comparison of anatomical access levels. Second, baseline pain severity and symptom duration were not balanced between groups despite non-significant baseline *p*-values in the original unadjusted analysis; all comparative findings therefore rely on baseline-adjusted models, and residual confounding by unmeasured variables cannot be excluded. Third, the NRS- and VAS-based estimates for arm and neck pain were directionally discordant at 6 months (Table 8); this discordance could not be resolved with the available data and further limits confidence in the comparative pain findings. Fourth, the retrospective design introduces inherent selection bias and limits the ability to establish causality, and treatment allocation was not randomized. Fifth, the absence of blinding may have introduced reporting bias, particularly for patient-reported outcome measures such as the NRS, VAS, and PGIC. Sixth, no formal correction for multiple comparisons was applied across the large number of secondary and safety outcomes, so secondary findings should be regarded as hypothesis-generating rather than confirmatory. Seventh, missing data (11 patients lost to follow-up by 6 months, and 8 additional patients excluded before analysis for incomplete data) were handled using available-case methods rather than multiple imputation; the extent and reasons for missingness are reported by group but the missing-at-random assumption cannot be verified. Eighth, a substantial proportion of baseline Neck Disability Index records could not be individually re-verified against source documentation, which limits confidence in the reported baseline NDI value specifically (see Section 3.3); other outcome measures did not show this issue. Ninth, the follow-up period was limited to 6 months, precluding assessment of long-term efficacy and durability of the observed benefit, and the reliance on subjective, patient-reported data across multiple instruments introduces further variability and potential measurement bias. Finally, the study did not include a placebo or conservative-treatment control group, which limits the ability to disentangle the specific contribution of the corticosteroid injection from the natural history of the condition, regression to the mean, and the effect of concurrent conservative therapies; the term “effectiveness” is used descriptively in this manuscript and should not be read as evidence of efficacy relative to no treatment. Tenth, outcome instruments were administered by the treating physician, a study coordinator, or a research nurse depending on center and visit, and the source records did not document a formal, standardized inter-rater training or certification protocol across centers, which may have introduced measurement variability that this dataset cannot quantify. Eleventh, concurrent non-pharmacological treatment during the follow-up period (e.g., continued or resumed physiotherapy) was not systematically captured in the source clinical documentation; concurrent pharmacological treatment is captured through the analgesic-consumption data, but the absence of complete non-pharmacological co-intervention data limits the ability to attribute observed changes to the injection alone.

Despite these limitations, the study has strengths that support its external validity. The multicenter design enhances generalizability across different clinical settings, and the inclusion criteria reflected real-world populations frequently encountered in interventional pain practice rather than a highly selected trial population. Core procedural elements (aseptic technique, fluoroscopic confirmation, use of a non-particulate steroid, 60-min post-procedural monitoring) were consistent across centers, although the injection level and injectate volume were center-specific rather than standardized; this distinction is discussed above and in the Limitations. The follow-up schedule (baseline, 3 months, 6 months) was part of routine post-procedural clinical care and was applied consistently, allowing characterization of both efficacy and safety outcomes across multiple validated instruments, but it was not a prospectively registered research protocol. Future prospective, ideally randomized, studies that vary injection level and injectate volume independently within the same center, with longer follow-up and a conservative-treatment or placebo comparator arm, would help to clarify the incremental benefit and the independent contribution of access level versus injectate volume, and could additionally incorporate structured, protocol-driven monitoring of blood pressure and blood glucose to better characterize the time course and clinical significance of the systemic effects identified here.

## 5. Conclusions

Both center-specific cervicothoracic interlaminar epidural steroid injection protocols (C7/T1–6 mL and T1/T2–8 mL) were associated with reductions in pain, improved functional outcomes, reduced analgesic consumption, and improved quality-of-life measures over six months in this retrospective cohort, and no catastrophic procedural complications were documented. However, this study cannot establish that either protocol is more effective than the other, that the two protocols are equivalent, or that injectate volume does not influence outcomes: injection level, injectate volume, and treatment center were confounded by design, baseline pain severity was not balanced between groups, the NRS- and VAS-based pain estimates were directionally discordant at 6 months, and the uncontrolled, non-randomized design precludes causal conclusions about comparative effectiveness. In baseline-adjusted analyses, the T1/T2–8 mL protocol was associated with a small but statistically significant disadvantage in arm- and neck-pain NRS at 3 and 6 months (Table 8); given the discordant VAS findings and the inseparability of level, volume, and center, this result should be interpreted cautiously rather than as proof that either protocol is inferior. Transient systemic effects—including blood pressure elevation and steroid-induced hyperglycemia—were common and clinically relevant. Post-procedural monitoring of blood pressure and glucose in patients with pre-existing hypertension or diabetes is consistent with prudent clinical practice, but this cohort alone is not a sufficient basis to establish it as a new standard of care; this recommendation should be considered alongside existing interventional pain guidelines.

## Figures and Tables

**Table 1:** Baseline demographic and clinical characteristics of patients undergoing cervicothoracic interlaminar epidural steroid injection at the C7/T1 and T1/T2 levels.

Characteristic	Group A (C7/T1) *n* = 87	Group B (T1/T2) *n* = 79	*p*-Value
Age, years	47.3 ± 10.7	48.2 ± 9.2	0.56
Sex, male, *n* (%)	46 (52.9)	42 (53.2)	1.00
Sex, female, *n* (%)	41 (47.1)	37 (46.8)	—
BMI, kg/m^2^	27.1 ± 3.2	26.8 ± 3.2	0.55
Symptom duration, months	5.6 ± 2.1	6.3 ± 2.1	0.03
Foraminal stenosis, 1 level, *n* (%)	56 (64.4)	54 (68.4)	0.71
Foraminal stenosis, 2 levels, *n* (%)	31 (35.6)	25 (31.6)	—
Pre-existing hypertension, *n* (%)	25 (28.7)	20 (25.3)	0.75
Pre-existing diabetes mellitus, *n* (%)	7 (8.0)	6 (7.6)	1.00

Data are presented as mean ± SD or *n* (%). BMI: body mass index. *p*-values were calculated using Student’s *t*-test for continuous variables and the χ^2^ test for categorical variables, comparing Group A (C7/T1) and Group B (T1/T2).

**Table 2:** Pain intensity (NRS and VAS) at baseline, 3 months, and 6 months, by treatment group.

Outcome	Baseline	3 Months	6 Months	*p* ^†^
NRS, Group A	8.7 ± 0.4	3.5 ± 0.4	4.1 ± 0.6	<0.001
NRS, Group B	9.2 ± 0.7	3.9 ± 0.5	4.9 ± 0.7	<0.001
VAS (mm), Group A	87 ± 4	37 ± 4	48 ± 7	<0.001
VAS (mm), Group B	90 ± 8	36 ± 5	43 ± 2	<0.001
Between-group *p*-value (A vs. B) ^‡^	0.51	0.38	0.44	—

Data are presented as mean ± SD. NRS: Numeric Rating Scale (0–10); VAS: Visual Analog Scale (0–100 mm). ^†^ *p*-value for within-group change from baseline (paired *t*-test). ^‡^ *p*-value for between-group comparison at each timepoint (Student’s *t*-test).

**Table 3:** Functional status, quality-of-life, and neuropathic pain outcomes (pooled cohort) at baseline, 3 months, and 6 months.

Outcome Measure (Pooled Cohort)	Baseline	3 Months	6 Months	*p*-Value
NDI (points)	49.1 ± 5.7	28.1 ± 4.5	31.4 ± 4.6	<0.001
SF-36 PCS	32.6 ± 2.7	47.2 ± 4.1	44.1 ± 4.3	<0.001
SF-36 MCS	35.9 ± 4.5	52.7 ± 5.2	49.2 ± 4.9	<0.001
EQ-5D index	0.46 ± 0.09	0.75 ± 0.03	0.63 ± 0.08	<0.001
DN4 (points)	6.2 ± 1.4	2.2 ± 0.7	3.1 ± 0.6	<0.001

Data are presented as mean ± SD. NDI: Neck Disability Index (0–50, higher = greater disability); SF-36 PCS/MCS: Short Form-36 Physical/Mental Component Summary (0–100, higher = better health); EQ-5D: EuroQol 5-Dimension utility index (0–1, higher = better health); DN4: Douleur Neuropathique en 4 Questions (0–10, ≥4 = neuropathic component). *p*-values refer to within-group change from baseline (paired comparison).

**Table 4:** Reduction in analgesic consumption relative to baseline, by medication class.

Medication Class	Reduction at 3 Months	Reduction at 6 Months
NSAIDs	65%	40%
Opioids (tramadol-based combinations)	60%	50%

Values represent the percentage reduction in average daily consumption relative to baseline; both reductions were statistically significant (*p* < 0.001, paired comparison).

**Table 5:** Distribution of Patient Global Impression of Change (PGIC) responses at 3 and 6 months.

PGIC Category	3 Months	6 Months
Much improved/very much improved	70% (116/166)	60% (93/155)
Moderately improved	20% (33/166)	20% (31/155)
Minimal or no improvement	10% (17/166)	20% (31/155)

PGIC: Patient Global Impression of Change, a single-item 7-point scale collapsed here into three clinically interpretable categories.

**Table 6:** Incidence of adverse events and transient systemic effects, overall and by treatment group, with between-group statistical comparison.

Adverse Event	Overall (*n* = 166)	Group A	Group B	*p*-Value
Dizziness/lightheadedness	30%	26%	34%	0.31
Transient upper-limb numbness	41%	40%	42%	0.88
Mild hypotension	16%	19%	12%	0.20
Transient disorientation	13%	9%	18%	0.12
Severe neurological/vascular complications	0%	0%	0%	1.00

Values represent the percentage of patients in each group experiencing the event at least once during the follow-up period. *p*-values compare Group A vs. Group B using Fisher’s exact test; none reached statistical significance. Absolute numerators (event/*n*): dizziness 50/166 overall (23/87 Group A vs. 27/79 Group B); upper-limb numbness 68/166 (35/87 vs. 33/79); hypotension 26/166 (17/87 vs. 9/79); disorientation 22/166 (8/87 vs. 14/79); severe neurological/vascular complications 0/166 (0/87 vs. 0/79).

**Table 7:** Cardiovascular and metabolic effects in patients with pre-existing hypertension or diabetes mellitus, and surgical outcomes at final follow-up.

Parameter	Subgroup, *n* (%)	Incidence
Pre-existing hypertension	45 (27.1)	—
Transient BP elevation (≤5 days post-injection)		78%
Temporary antihypertensive adjustment required		40%
Pre-existing diabetes mellitus	13 (7.8)	—
Transient hyperglycemia (≤7 days)		90%
Additional glycemic treatment required		60%
Surgical treatment at final follow-up (27/166; 14/87 C7/T1 vs. 13/79 T1/T2)	166	16%

BP: blood pressure. Percentages for hypertension and diabetes mellitus are calculated relative to the whole cohort (*n* = 166); incidences of transient BP elevation, antihypertensive adjustment, hyperglycemia, and glycemic treatment adjustment are calculated within the respective subgroup.

**Table 8:** Baseline-adjusted between-group differences (ANCOVA), group × time interaction (GEE), and multivariable logistic regression for treatment response (T1/T2–8 mL vs. C7/T1–6 mL protocol).

Outcome	Comparison/Model	Estimate	95% CI	*p*-Value
NRS, arm pain	3 months (ANCOVA, adj. diff.)	+0.41	0.21, 0.61	<0.001
NRS, arm pain	6 months (ANCOVA, adj. diff.)	+0.66	0.35, 0.97	<0.001
NRS, arm pain	Group × time (GEE)	+0.15	0.02, 0.28	0.024
NRS, neck pain	3 months (ANCOVA, adj. diff.)	+0.55	0.30, 0.79	<0.001
NRS, neck pain	6 months (ANCOVA, adj. diff.)	+0.76	0.40, 1.13	<0.001
NRS, neck pain	Group × time (GEE)	+0.19	0.03, 0.34	0.019
VAS, arm pain	3 months (ANCOVA, adj. diff.)	−0.69	−2.61, 1.23	0.48
VAS, arm pain	6 months (ANCOVA, adj. diff.)	−5.54	−7.93, −3.14	<0.001
VAS, arm pain	Group × time (GEE)	−3.90	−5.18, −2.62	<0.001
VAS, neck pain	3 months (ANCOVA, adj. diff.)	−0.33	−2.79, 2.13	0.79
VAS, neck pain	6 months (ANCOVA, adj. diff.)	−5.58	−8.50, −2.66	<0.001
NDI (raw, 0–50)	3 months (ANCOVA, adj. diff.)	−0.73	−2.65, 1.19	0.46
NDI (raw, 0–50)	6 months (ANCOVA, adj. diff.)	−0.68	−2.81, 1.44	0.53
SF-36 PCS	3 months (ANCOVA, adj. diff.)	+0.58	−1.18, 2.35	0.51
SF-36 PCS	6 months (ANCOVA, adj. diff.)	+0.32	−1.61, 2.25	0.74
SF-36 MCS	3 months (ANCOVA, adj. diff.)	+0.13	−2.05, 2.31	0.91
SF-36 MCS	6 months (ANCOVA, adj. diff.)	−0.82	−3.05, 1.41	0.47
EQ-5D index	3 months (ANCOVA, adj. diff.)	−0.01	−0.02, 0.00	0.18
EQ-5D index	6 months (ANCOVA, adj. diff.)	+0.02	−0.02, 0.05	0.35
DN4 (points)	3 months (ANCOVA, adj. diff.)	−0.25	−0.55, 0.05	0.10
DN4 (points)	6 months (ANCOVA, adj. diff.)	−0.09	−0.37, 0.18	0.50
Responder ≥ 50% NRS-arm reduction, 3 mo (logistic regression)	Protocol, T1/T2 vs. C7/T1 (OR)	0.18	0.04, 0.82	0.026
	Age, per year (OR)	0.97	0.91, 1.04	0.43
	Symptom duration, per month (OR)	1.02	0.77, 1.35	0.89
	Stenotic levels, per level (OR)	0.67	0.19, 2.39	0.53
	DN4 score (OR)	0.89	0.60, 1.34	0.58
	Center 2 vs. Center 1 (OR)	0.49	0.11, 2.27	0.36
	Center 3 vs. Center 1 (OR)	3.15	0.41, 24.05	0.27

GEE = generalized estimating equations (exchangeable working correlation); coefficients represent the group × time interaction term. OR = odds ratio; CI = confidence interval. NDI = Neck Disability Index (raw 0–50 scale); see Section 3.3 and Limitations regarding baseline NDI data-quality concerns. Positive adjusted differences for NRS/VAS indicate higher (worse) pain in the T1/T2–8 mL group relative to C7/T1–6 mL; negative values indicate lower (better) pain. All models adjusted for baseline outcome value (where applicable), age, symptom duration, number of stenotic levels, DN4 score, and treatment center. The directionally discordant NRS versus VAS estimates for arm and neck pain at 6 months are discussed in Section 4 (Discussion) and the Limitations.

## Data Availability

The original contributions presented in this study are included in the article/Supplementary Materials. Further inquiries can be directed to the corresponding author.

## Supplementary Materials

The following supporting information can be downloaded at: https://www.mdpi.com/article/10.3390/jcm15155900/s1, Table S1: Number of patients treated with each injection protocol, by participating center; Table S2: Reasons for non-inclusion of screened patients, by center (n = 48 excluded of 214 screened); Table S3: Patients with data available at each assessment timepoint, by treatment protocol; Table S4: STROBE Statement checklist for cohort studies.

## Abbreviations

The following abbreviations are used in this manuscript:

BMI	Body Mass Index
BP	Blood Pressure
CONSORT	Consolidated Standards of Reporting Trials
DM	Diabetes Mellitus
DN4	Douleur Neuropathique en 4 Questions
EQ-5D	EuroQol 5-Dimension
ESI	Epidural Steroid Injection
HTN	Hypertension
LOR	Loss of Resistance
MCS	Mental Component Summary
MRI	Magnetic Resonance Imaging
NDI	Neck Disability Index
NRS	Numeric Rating Scale
NSAID	Nonsteroidal Anti-Inflammatory Drug
PCS	Physical Component Summary
PGIC	Patient Global Impression of Change
SD	Standard Deviation
SF-36	Short Form-36 Health Survey
VAS	Visual Analog Scale
