# Supplementary material for: Clinical Outcomes of Two Institution-Specific Cervicothoracic Interlaminar Epidural Steroid Injection Protocols (C7/T1–6 mL vs. T1/T2–8 mL) in Cervical Radiculopathy: A Multicenter Retrospective Cohort Study
Source: J Clin Med. 2026 Jul 28;15(15):5900. doi: 10.3390/jcm15155900 (PMC13467484; doi:10.3390/jcm15155900)
Supplement: Supplementary file 1 [file jcm-15-05900-s001.zip › jcm-4453856-supplementary.pdf]

Supplementary Materials

Table S1. Number of patients treated with each injection protocol, by participating center.

Center	C7/T1–6 mL, n	T1/T2–8 mL, n	Total, n
Neurolocus (Warszawa)	60	10	70
Szpital Matki Bożej Nieustającej Pomocy (Wołomin)	27	25	52
Szpital Powiatowy im. R. Surmy (Czarnków)	0	44	44
Total	87	79	166

Two of the three participating centers (Neurolocus and the Wołomin hospital) used both injection protocols; the Czarnków hospital used only the T1/T2–8 mL protocol during the study period. Because of this distribution, treatment protocol, injection level, injectate volume, and treatment center are not fully statistically separable in this dataset — see Limitations in the main text.

Table S2. Reasons for non-inclusion of screened patients, by center (n = 48 excluded of 214 screened).

Center	Did not meet eligibility criteria, n	Declined participation/procedure, n	Incomplete data, n
Neurolocus (Warszawa)	13	3	1
Szpital Matki Bożej Nieustającej Pomocy (Wołomin)	8	1	4
Szpital Powiatowy im. R. Surmy (Czarnków)	10	5	3
Total	31	9	8

"Did not meet eligibility criteria" includes absence of MRI-confirmed radiculopathy, symptom duration under six weeks, prior cervical surgery at the index level, and contraindications to corticosteroid administration. "Incomplete data" reflects records with missing baseline or follow-up documentation sufficient to preclude inclusion.

Table S3. Patients with data available at each assessment timepoint, by treatment protocol.

**Timepoint**	**C7/T1–6 mL (n = 87)**	**T1/T2–8 mL (n = 79)**	**Total (n = 166)**
Baseline	87 (100%)	79 (100%)	166 (100%)
3 months	87 (100%)	79 (100%)	166 (100%)
6 months	81 (93.1%)	74 (93.7%)	155 (93.4%)

No patient had a fully missing baseline or 3-month assessment. Eleven patients (6/87 in the C7/T1–6 mL group; 5/79 in the T1/T2–8 mL group) were lost to follow-up by 6 months and are excluded from 6-month analyses (available-case approach; see Section 2.5 of the main text). Among patients with a recorded visit, no individual outcome variable (NRS, VAS, DN4, NDI, SF-36, EQ-5D) had missing values at that visit.

Table S4. STROBE Statement checklist for cohort studies

**Section**	**Item**	**Recommendation (STROBE — cohort studies)**	**Where addressed in the manuscript**
Title and abstract	1a	Indicate the study's design with a commonly used term in the title or the abstract.	Title: "...A Multicenter Retrospective Cohort Study" states the design explicitly.
	1b	Provide an informative and balanced summary of what was done and what was found.	Abstract (Methods/Results/Conclusions) states the design limitations and reports the adjusted findings rather than only unadjusted comparisons.

Introduction	2	Explain the scientific background and rationale for the investigation being reported.	Introduction, with cervical-specific literature on injectate spread, C7/T1 vs. T1/T2 comparative data, and current guidelines.
	3	State specific objectives, including any prespecified hypotheses.	Final paragraph of the Introduction: states the study is retrospective and multicenter, and that no directional hypothesis was prespecified (exploratory study).
Methods	4	Present key elements of study design early in the paper.	Section 2.1; analytic model detailed in Section 2.5.
	5	Describe the setting, locations, and relevant dates, including periods of recruitment, exposure, follow-up, and data collection.	Section 2.1; center-level enrollment by protocol is reported in Table S1 (Supplementary Materials).
	6a	Give the eligibility criteria, and the sources and methods of selection of participants.	Section 2.2 (retrospective identification of consecutive eligible patients from institutional records).
	6b	For studies with exposed and unexposed groups: give eligibility criteria for each group.	Exposure = center-specific protocol (injection level + volume); described in Sections 2.1–2.3 and in Limitations. Center-by-protocol enrollment counts in Table S1.
	7	Clearly define all outcomes, exposures, predictors, potential confounders, and effect modifiers.	Section 2.5 gives the DAG-based confounder list (age, symptom duration, baseline pain severity, number of stenotic levels, DN4, treatment center).
	8	For each variable of interest, give sources of data and details of methods of assessment.	Section 2.4; questionnaire validation references (NDI, SF-36, EQ-5D Polish versions) are in the bibliography.
	9	Describe any efforts to address potential sources of bias.	Section 2.5: standardized mean differences reported for baseline balance instead of relying on p-values alone; multivariable adjustment for DAG-selected covariates.
	10	Explain how the study size was arrived at.	Section 2.5 states that all consecutive eligible patients treated during the study period were included and that no prospective sample-size calculation was performed; a precision/CI-width analysis is

			reported instead of post-hoc power.
	11	Explain how quantitative variables were handled; describe grouping and why.	Section 2.5 describes the ANCOVA and GEE approaches.
	12a–e	Statistical methods, including those for confounding, missing data, sampling, and sensitivity analyses.	Section 2.5: DAG-informed multivariable logistic regression, GEE (group×time interaction), ANCOVA; handling of missing data (available-case); explicit statement that no correction for multiplicity was applied to secondary/exploratory outcomes.
Results	13a–c	Numbers of individuals at each stage of the study; reasons for non-participation.	Section 3.1; STROBE-style flow diagram (Figure 1); full breakdown of the 48 non-included patients by reason and center in Table S2.
	14a–c	Descriptive data on characteristics and exposures; numbers with missing data.	Table 1; missing-data-by-timepoint reported in Table S3.
	15	Report numbers of outcome events or summary measures over time.	Tables 2–7.
	16a–c	Give unadjusted and, where applicable, confounder-adjusted estimates with precision; report category boundaries; consider absolute risk.	Section 3.9 and Table 8 report baseline-adjusted between-group differences (ANCOVA), group×time interaction (GEE), and the multivariable logistic regression model directly in Results, with 95% CIs.
	17	Report other analyses done — e.g., subgroup and sensitivity analyses.	Responder analysis (≥50% arm-pain NRS reduction) via multivariable logistic regression, reported in Table 8.
Discussion	18	Summarize key results with reference to study objectives.	Discussion opening paragraph; avoids overclaiming — differences are not attributed to a single design factor given the confounding between level, volume, and center.
	19	Discuss limitations, sources of bias, and their likely direction and magnitude.	Limitations section: injection level, injectate volume, and center are confounded by design; baseline imbalance; the NRS–VAS directional discordance at 6 months; no multiplicity correction; missing data; the Neck Disability Index data-quality concern; absence of a control arm.
	20	Give a cautious overall interpretation considering objectives, limitations, and multiplicity of analyses.	Discussion and Conclusions: the adjusted arm/neck-pain NRS finding is explicitly flagged as not

			proof that either protocol is inferior, given the discordant VAS results and the inseparability of level, volume, and center.
	21	Discuss the generalizability (external validity) of the study results.	Strengths paragraph notes that procedural technique (not injection level/volume) was standardized across centers, and that follow-up was routine clinical care rather than a prospectively registered protocol.
Other information	22	Give the source of funding and the role of funders.	Funding statement: no external funding.
